# Impact of country of birth on genetic testing of metastatic lung adenocarcinomas in France: African women exhibit a mutational spectrum more similar to Asians than to Caucasians

**DOI:** 10.18632/oncotarget.15132

**Published:** 2017-02-07

**Authors:** Raphael Saffroy, Jean-François Morère, Nelly Bosselut, Pasquale F. Innominato, Jocelyne Hamelin, Jean Trédaniel, Sophie Masse, Véronique Dussaule-Duchatelle, André Balaton, Pierre Validire, Catherine Guettier, Mohamed Bouchahda, Antoinette Lemoine

**Affiliations:** ^1^ AP-HP, GH Paris-Sud, Hôpital Paul Brousse, Department of Biochiemistry and Oncogenetics, Platform Oncomolpath/INCa, Villejuif, France; ^2^ INSERM UMR-S 1193, Université Paris-Sud, Université Paris-Saclay, Villejuif, France; ^3^ AP-HP, GH Paris-Sud, Hôpital Paul Brousse, Department of Medical Oncology, Villejuif, France; ^4^ INSERM UMR-935, Université Paris-Sud, Hôpital Paul Brousse, Villejuif, France; ^5^ Cancer Chronotherapy Unit, Cancer Research Centre, Warwick Medical School, University of Warwick, Coventry, United Kingdom; ^6^ Department of Oncology, Queen Elizabeth Hospital Birmingham NHS Foundation Trust, Birmingham, United Kingdom; ^7^ Hôpital Saint Joseph, Department of Respiratory Medicine, Université Paris 5, Paris, France; ^8^ Groupe Hospitalier Nord Essonne, Department of Pathology, Longjumeau, France; ^9^ Hôpital Saint Joseph, Department of Pathology, Paris, France; ^10^ ACP Bievres-les Ulis, Department of Pathology, Les Ulis, France; ^11^ Institut Mutualiste Montsouris, Department of Pathology, Paris, France; ^12^ AP-HP, GH Paris-Sud, Hôpital Paul Brousse, Department of Pathology, Platform Oncomolpath/INCa, Villejuif, France; ^13^ Ramsay-GDS Clinique du Mousseau, Department of Medical Oncology, Evry, France

**Keywords:** NSCLC, genetic testing, genotype, EGFR, ERBB2

## Abstract

**Background:**

Limited data are available on the prevalence of oncogenic driver mutations in Caucasian populations, and especially in Europeans.

**Aim:**

To evaluate the targetable mutational spectra in unselected patients with lung adenocarcinoma in routine clinical practice from several French hospitals, using the same molecular platform.

**Patients and Methods:**

Samples from 2,219 consecutive patients with histologically-proven advanced lung adenocarcinoma were centrally analysed at a referenced and certified diagnostic platform in order to test for activating and resistance mutations in *EGFR*, *KRAS*, *BRAF*, *ERBB2* and *PI3KCA*. Demographic and clinical features were retrieved from the medical charts. Multivariate binary logistic regression was used to determine the independent predictive factors for the occurrence of specific mutations, in the whole study population or in selected subgroups.

**Findings:**

The overall respective incidence of *EGFR*, *KRAS*, *BRAF*, *ERBB2* and *PI3KCA* mutations was 10.5%, 0.9%, 25%, 1.5%, 2.1% and 1.4%, in our study sample including 87.4% white Caucasians, 10.8% Africans and 1.8% Asians; 60.6% men, 30.7% never smoker (median age: 68.3 years). Ethnicity was an independent predictor for EGFR, KRAS and ERBB2 gene abnormalities. In all cases, a significantly higher prevalence of targetable *EGFR* and *ERBB2*, and a lower prevalence of resistance *KRAS* mutations were observed in African women as compared to African men or Caucasians.

**Conclusions:**

In real life conditions of routine genetic testing, we have identified subsets of patients with specific targetable activating somatic mutations according to ethnicity, who could preferentially benefit from anti-*EGFR* and anti-*ERBB2* targeted therapies.

## INTRODUCTION

The identification of EGFR mutations as activating mutations conferring sensitivity to anti-EGFR therapies like gefitinib or erlotinib was firstly reported in 2004 by 2 groups studying American and Asian patient populations [1–3]. They also observed that EGFR mutations in exons 18 to 21 were more prevalent in Japaneses (30%) as compared to Caucasians (only 7%). In 2009, the IRESSA Pan-Asia Study (IPASS), which compared the EGFR tyrosine kinase inhibitor (TKI), gefitinib, with carboplatin-paclitaxel cytotoxics, was conducted on patients of Asian descent with adenocarcinoma, as a mutation-enriching strategy in the study population. The trial demonstrated that gefitinib was associated with a 12-month progression-free survival rate of 24.9%, in comparison to 6.7% in the chemotherapy arm [4]. This key study has authorized the use of gefitinib as first-line therapy in patients whose metastatic adenocarcinoma carries EGFR mutations.

Women, and especially never smokers, also exhibit a higher EGFR mutational frequency, especially in Asian never smokers, with a rate ranging between 48% and 75.3% [5–8].

A number of institutions worldwide have integrated EGFR molecular profiling into routine lung cancer diagnosis to personalize treatment decisions, and especially the use of TKIs [9]. The vast amount of genomic information has shed light on high variations in EGFR mutational frequencies, ranging from 7% to 40.1% within the Asian population [10–17]. EGFR mutation frequency was lower in populations not of Asian origin, with a high genetic heterogeneity around the world [18]. Indeed, in Latin populations, either in Spain or in Latin America, the frequency of EGFR mutations in NSCLC has been reported to vary from 16.6% to 37% [19, 20]. In America where Latin-Americans or African-Americans with NSCLC are included in the screening for EGFR, the frequency of EGFR testing is approximately 25% [21–26]. In Europe, except for Spain, the frequency of EGFR mutations is the lowest reported, in approximately 10% of metastatic or advanced lung adenocarcinomas [27–32]. Nonetheless, only limited data on the prevalence of EGFR mutations are available in Caucasian populations, and especially in Europeans. Moreover, there are few data on the mutation rates of targetable oncogenic *BRAF*, *ERBB2*, *KRAS* or *PI3KCA* mutations across worldwide populations [33–35]. Corresponding information regarding the African population remains deficient except for African-Americans [21–26], although North Africans represent an important proportion of NSCLC patients daily treated in French hospitals.

The aim of our study was to analyze the mutational rate of *EGFR*, *KRAS*, *BRAF*, *ERBB2* and *PI3KCA* in a large cohort of 2,219 unselected French patients presenting in routine clinical practice for first line treatment decision of metastatic or recurrent lung adenocarcinoma.

## RESULTS

### Characteristics of the French cohort of 2,219 patients with lung adenocarcinoma testing for EGFR mutations

All tumours were adenocarcinomas, and all patients had advanced stage disease. Eighty percent of the samples analysed were biopsies from lung tumors (through transbronchial fibroscopy or percutaneous scan-guided biopsy); the remaining 20% were surgical specimens or biopsies from metastatic lesions, mainly in supradiaphragmatic lymph nodes.

Patients were diagnosed at a median age of 68.3 years (range, 26.6 to 93.7 years; standard deviation, 11.1 years). There was a higher proportion of men (60.6% men *vs* 39.4% women) and of tobacco-exposed individuals (48.2% current smokers; 21.1% former smokers; 30.7% never smokers), with a median of 20 packs year for the smokers (Table 1).

**Table 1:** Clinical and demographic features of the whole study sample (N=2, 219)

Feature		N	%
**Gender**	Male	1,345	60.6
	Female	874	39.4
**Smoking status**	Never	633	28.5
	Former	434	19.6
	Current	993	44.7
	Unknown	159	7.2
**Continent**	Europe	1,940	87.4
	Asia	39	1.8
	Africa	240	10.8
**Age**	Median	68.3	
	1^st^-3^rd^ quartile	61.0-76.5	
	Range	26.6-93.7	

Among the 2,219 consecutive patients that constitute our study population, most of them were Caucasian (1,940 patients; 87.4%); those of African ancestry (240 patients; 10.8%) were more common than those of Asian origin (39 patients; 1.8%).

### Mutational spectrum of the study population

**Figure 1:**
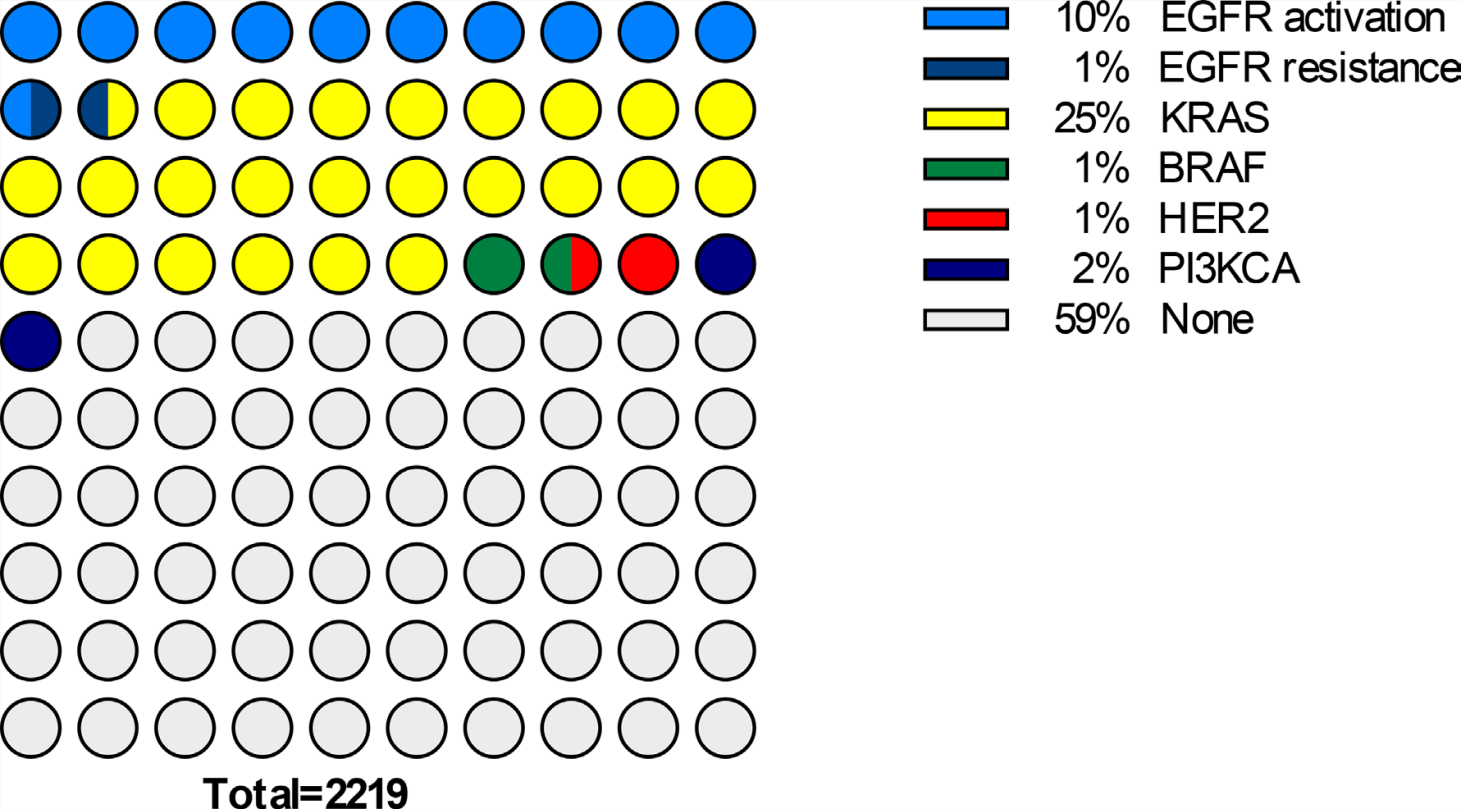
Chart representing the proportions of patients with each mutation of the spectrum analysed, within the whole study sample (N=2, 219)

*KRAS* mutations were altogether the most common genetic abnormalities observed (Figure 1). Indeed, a fourth of the samples was mutated for *KRAS* exon 2 or 3 (95% Confidence Limits: 23.2%-26.8%), whereas 58.9% of the samples (95% CL: 56.9%-61.0%) did not show any genetic alteration within our panel. The observed prevalence of activating EGFR mutations (activating common and uncommon mutations in exon 18 to 21) was 10.5% (95% CL: 9.2%-11.8%); EGFR resistance mutations in exon 20 were present in only 0.9% of the samples (95% CL: 0.5% - 1.3%). Respective prevalence of BRAF, *PI3KCA* or *ERBB2* alterations was 1.5% (1.0%-2.0%), 2.1% (1.5%-2.7%) and 1.4% (0.9%-1.9%).

### Frequency of EGFR mutations

Activating *EGFR* mutations have to date the most prominent therapeutic role in the clinical management of NSCLC. The independent predictors of activating *EGFR* mutations included gender, smoking status, age and ethnic origin (Table 2). Thus, activating *EGFR* mutations were almost thrice more common in women (17.7%) than in men (6.1%), and in non-smokers (74.5%) than in smokers (current: 26.2%; former: 25.7%). Older patients were also more likely to have a tumor with activating *EGFR* mutation, with a prevalence per age quartiles increasing from 7.7% in younger patients (< 61 years) to 15.6% in older patients (> 76 years). Patients of Asian descent had the highest prevalence of activating EGFR mutations (36.1%), in comparison to Caucasians (10.2%) or Africans (9.3%). However, the prevalence of mutated *EGFR* according to gender was more dissimilar in African patients (males: 5.2%; females: 29.3%) than in Caucasians (5.7% in men and 16.8% in women).

**Table 2:** Independent predictive factors for *EGFR* activating mutation, *KRAS* mutation and *ERBB2* endoduplication in the whole study sample (multivariate analysis)

Parameter		HR	95% CL	*p*
***EGFR* activating mutation**				
**Continent**				**<0.0001**
	Europe	1		
	Asia	5.65	2.67-12.0	<0.0001
	Africa	1.27	0.77-2.10	0.35
**Smoking status**				**<0.0001**
	Never	1		
	Former	0.12	0.04-0.32	<0.0001
	Current	0.12	0.06-0.27	<0.0001
**Gender**				**<0.0001**
	Male	1		
	Female	3.50	2.59-4.71	
**Age**		1.03	1.02-1.05	<0.0001
***KRAS* mutation**				
**Continent**				**0.01**
	Europe	1		
	Asia	0.24	0.07-0.79	0.019
	Africa	0.59	0.41-0.84	0.004
**Age**		0.986	0.977-0.994	0.01
***ERBB2* endoduplication**				
**Continent**				**<0.0001**
	Europe	1		
	Asia	9.96	2.77-35.8	<0.0001
	Africa	4.32	1.80-10.4	0.001

#### Subtypes of EGFR mutations

Subtypes of EGFR mutations were overall distributed as follows: L858R was observed in 63 patients (2.8%), del19 in 81 patients (3.7%), T790M and other rare ones in 13 (0.6%) patients each. However, the incidence of each mutation differed according to ethnicity. Thus, the ratio del19/L858R was 1.31 in Europeans, 2.00 in Asians and 0.86 in Africans, respectively. Additionally, the relative frequency of T790M among all EGFR mutations was 5.6% in Europeans, 14.3% in Asians, and 21.1% in Africans.

### Frequency (prevalence) of KRAS mutations

*KRAS* mutations were the most commonly encountered genetic alterations in our study populations, and age and ethnic origin independently predicted for their occurrence (Table 2). Thus, their prevalence dropped from 28.1% in younger patients to 20.3% in older ones. Caucasians had the highest prevalence of *KRAS* mutations (26.3%), whilst Asians had the lowest (7.7%); Africans displayed an intermediate prevalence (17.2%).

The prevalence of mutated *KRAS* according to gender was most similar in patients with Caucasian background (males: 25.2% and females: 28.2%), intermediate in Africans (18.8% in men and 11.9% in women) and most dissimilar in Asians (males: 13.0%; females: 0%).

### Frequency of BRAF, PI3KCA and ERBB2 mutations

Amidst the least frequent mutations, no independent predictive factor was identified for *BRAF* mutations, whereas increasing age had a positive predictive value for the occurrence of *PI3KCA* mutations (HR: 1.048; 95% CL: 1.018 to 1.078; p=0.002). Interestingly, ethnic origin was the only independent predictive factor for *ERBB2* mutations (Table 2). Indeed, this genetic alteration occurred in 8.1% of Asian patients, in 3.3% of African ones, and in only 1.0% of those of Caucasian descent.

The prevalence of mutated *ERBB2* according to gender was rather similar in patients with Asian (men: 8.7%; women: 7.7%) or Caucasian (0.9% and 1.3%) background, and fairly different in African patients (males: 2.1%; females: 9.3%).

### Ethnic demographics

The observation that the mutational phenotype in patients from Africa appeared intermediate between that of Europeans and that of Asians prompted us to explore more in depth the effect of ethnic origin on the mutational phenotype of NSCLC patients treated in France. Hence, we focused on the subgroup of patients from Africa, which is often underrepresented and overlooked in Western studies in NSCLC, despite it accounts for a fairly substantial proportion of patients treated for NSCLC (in our study, 10.8%).

The clinical and demographic features of the 240 patients from Africa are summarized in Table 3. Thus, as compared to the other ethnic subgroups, African patients were more commonly males (81.7% versus 58.1%; p <0.0001) and smokers (never: 14.3% versus 35.1%; p=0.018), whereas no age difference with the rest of the patients was observed (median: 68.9 versus 68.2 years).

**Table 3:** Clinical and demographic features of the subset from Africa (N=240)

Feature		N	%
**Gender**	Male	196	81.7
	Female	44	18.3
**Smoking status**	Never	34	14.3
	Former	41	17.1
	Current	165	68.6
**Age**	Median	68.9	
	1^st^-3^rd^ quartile	59.6-75.1	
	Range	38.1-92.0	

**Figure 2:**
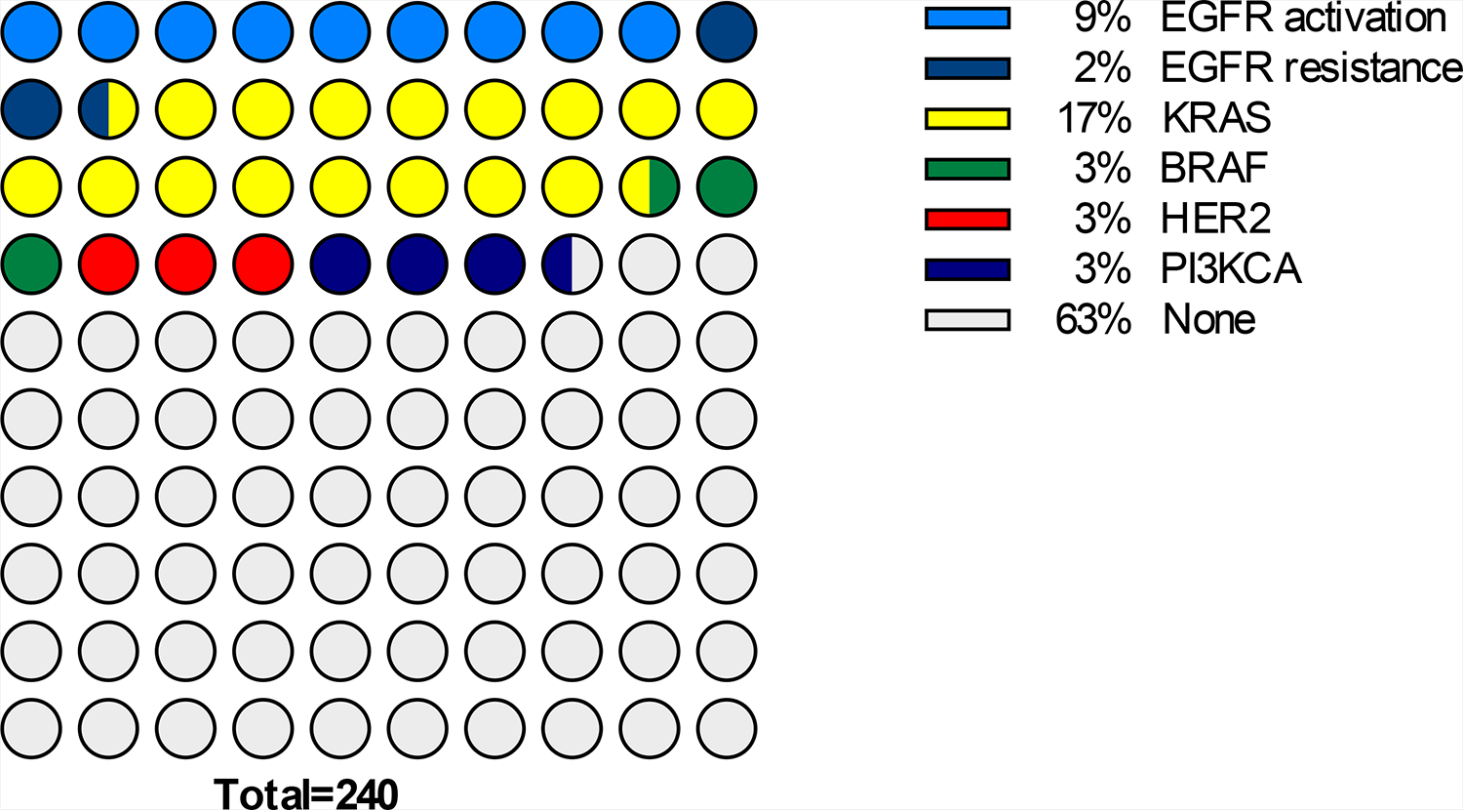
Chart representing the mutational spectrum of the subset of patients of African descent (N=240)

The mutational spectrum of African patients is shown in Figure 2. This ethnic group displayed a higher prevalence of *EGFR* resistance mutations (p=0.014), yet not of *EGFR* activating mutations (p=0.53), a higher prevalence of ERBB2 alterations (p=0.007); a lower prevalence of *KRAS* (p=0.004) and *BRAF* (p=0.045) mutations, and a not significant difference in *PI3KCA* (p=0.14).

However, subgroup analysis according to gender suggested that African women have a peculiar mutational spectrum, with an independently higher risk of presenting *EGFR* activating (HR: 2.05; 95% CL: 1.02-4.12; p=0.044) and *EGFR* resistance mutations (HR: 4.16; 95% CL: 1.17-14.77; p=0.028). Additionally, African women displayed a notably higher risk of having cancers harbouring *ERBB2* mutations (HR: 8.01; 95% CL: 2.41-26.68; p=0.001), and a lower risk of *KRAS* mutations (HR: 0.34; 95% CL: 0.13-0.89; p=0.027).

**Figure 3:**
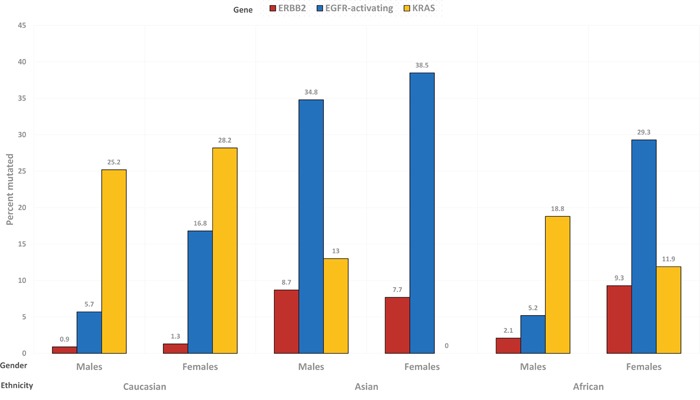
Prevalence of ERBB2 (red), EGFR-activating (blue) and KRAS (yellow) mutations, in the subgroups determined by gender and ethnicity

The observed prevalence of the main mutations, in man and women from the three ethnic groups is depicted in Figure 3.

## DISCUSSION

We examined 2,219 unselected patients treated in hospitals in Paris and suburbs (France) for metastatic or recurrent lung adenocarcinoma, who were systematically tested for *EGFR* and other targetable driver mutations in the daily clinical setting. We found, similarly to other groups, an overall rate of activating *EGFR* mutations of 10.5% [18, 32]. *KRAS* was the most prevalent mutation with 25.0% of positive cases, whereas *BRAF*, *ERBB2* and *PI3KCA* mutations were relatively uncommon, with 1.5, 1.4, and 2.1% mutational rates, respectively. Gender, smoking habits, but also ethnicity, alone and combined, appeared to influence these figures. Indeed, in the subpopulation of migrants from Africa (1^st^ or 2^nd^ generations), the molecular phenotype appeared peculiar, with prominent gender-specific differences. For example, *EGFR* activating and resistance mutation rates were as high as 29.3% and 26.7% in African women, respectively, whilst respective rates being as low as 5.2% and 7.1% in African men. Similarly, and very interestingly, *ERBB2* was mutated in 9.3% of African women, and in only 2.1% of men from Africa. Overall, rates of *PI3KCA* mutations were more frequent in females than in males from Africa, although not to a significant extent. These data could suggest that African women have a more genetically instable phenotype, even though *KRAS* was not more frequently mutated in women than in men from Africa. However, KRAS was altogether less frequently mutated in patients from Africa (17%) than in the global population (25%).

In Europe, the first reports of lung-cancer–specific *EGFR* mutations were described in Italy, and showed *EGFR* mutations in 10% of 375 lung adenocarcinomas [27], whereas in Spain it was described a rate of *EGFR* mutations of 16.6% [19]. Since the development of systematic *EGFR* mutational status testing in metastatic adenocarcinomas to identify patients who can benefit from first line TKIs, several reports have shown that the *EGFR* mutations prevalence is approximately 10% in Northern European populations [27–32]. In North America, several recent reports on the *EGFR* testing show a generally higher prevalence (approximately 25%), but most of them include a variety of heterogeneous racial groups and smoking habits [8, 26]. Similarly, it has been reported that the prevalence of *EGFR* mutations is 2 to 3-fold higher in Asians, with differences between Eastern and Western regions observed [10–17]. In the French population studied here, the subgroup from Asia was relatively underrepresented. However, in this ethnic subgroup counting 39 patients, we observed the expected higher *EGFR* mutational rate as compared to that in Caucasians, with a prevalence of 36.1%. Similarly, we observed, as already previously reported, that women and non-smokers exhibited more frequently *EGFR* activating mutations.

Thus, the analysis per ethnicity in our French unselected population showed similar *EGFR* mutational rates between Africans and Caucasians, despite an almost halved proportion of never smokers and a double proportion of males in our subgroup of patients of African descent.

Contrariwise, the *KRAS* mutational rate of Africans (17.2%) was intermediate between the highest one in Caucasians (26.3%) and the lowest one in Asians (7.7%). These results corroborate previous results showing that KRAS mutations are less common in Asians compared to Caucasians [6, 36, 37].

Similarly, the prevalence of *ERBB2* mutation in patients of African origin (3.3%) was intermediate between that observed in Asian (8.1%) and Caucasian (1.0%) patients.

However, although the global prevalence of *ERBB2* mutations was 1.4%, figure consistent with recent publications [38–41], the mutation rate of 3.3% observed here in the African population reaches an interesting threshold to be systematically screened for, in this selected subgroup of patients. This is even more clinically relevant since recent studies demonstrated a significant therapeutic benefit of trastuzumab (an anti-ERBB2 mAb) and of newer TKIs, such as afatinib and neratinib, which target both EGFR and ERBB2, in selected patients with lung adenocarcinoma [38, 42–46].

The prevalence of *EGFR* mutation has been recently reported in African populations. Errihani et al. [47] have studied a Moroccan population of 137 patients with an overall frequency of the *EGFR* mutations of 21%. However, they reported a high incidence of *EGFR* mutations with an unusual incidence of exon 21 L858R/exon 19 frameshift base pair deletions or exon 20 mutations. Nonetheless, they tested tumors on the French continent in a single laboratory, and did not mention to be from consecutive patients. We could therefore suspect a biased selection of patients presenting in Moroccan university hospitals who have then benefited abroad from oncogenetic testing. Other reports in American studies analysing African Americans subpopulations show a lower frequency of somatic driver mutations than in Caucasians [21]. We observed, nevertheless, that patients of African descent have a relatively higher incidence of T790M mutations (more than 4-fold), and a shift towards more frequent L858R rather than del19 (1.5-fold), in comparison with European patients.

Interestingly, some studies in Latin America populations report higher incidence of *EGFR* mutations than that found in Europe. A recent study shows an overall frequency of EGFR mutations of 26% in Latin America (from 14.4% in Argentina to 31% in Costa Rica) [48]. Interestingly, in Latin America, the frequency of KRAS mutations was as low as 14%. These results corroborate the importance of racial differences in the oncogenic driver gene mutations and could explain the higher incidence of *EGFR* mutations in Spain than in other European countries such as France or Germany. In a recent report, different EGFR and KRAS mutational ratio across South America was reported, and, allegedly, the migration of population through the Bering Strait could account for this observation [49].

Such migrations could explain the variations in the incidence of driver oncogene mutations across populations, and shed light on the importance of ethnic background to favour some genetic testing in subpopulations with the aim of increasing the number of patients who could benefit from targeted therapies.

Ethnic minorities in France, similarly to the rest of the European Community, tend to have immigrated mostly from Africa, rather than from Asia, contrarily to what observed in North America or Oceania. As a consequence, our unselected consecutive population included a higher proportion of Africans (10.8%) than of Asians (1.8%). Furthermore, smoking habits, especially in women, are probably different between ethnic groups, with 1^st^ or 2^nd^ generation North-Africans females being unlikely smokers. This social context could influence the mutational spectra of NSCLC diagnosed in French metropolitan areas in different ethnic subgroups and genders.

Our study, albeit large, has several limitations. Firstly, the assignment of ethnicity in this study was potentially prone to contain mistakes, yet it was based on the objective evaluation of birth places of the patient and of his/her parents. Indeed, French legislation forbids to record the ethnic origin of the patients. Hence, we were compelled to assume the ethnicity of each patient by considering the Country of birth. However, in the globalized world context, we fully acknowledge that the birthplace does not necessarily guarantee the ethnicity of the person. Nevertheless, by estimating the ethnic origin of each patient as that of the largest proportion of the population in the Country of birth, we can presume to have minimised the mismatches, albeit doubtlessly present, in our study. Moreover, our study population included a relatively low proportion of non-white Europeans, despite the multicultural milieu observed in the greater Paris region nowadays. In particular, given the relatively small number of patients of Asian descent in our study, the estimation of the frequency of rare mutations across subgroups could be rather imprecise. The rather low proportion of non-Europeans could be due to the age distribution, with several retired immigrants having decided to move back to their home Countries, while the younger ones remaining in France have not yet developed lung cancer. Furthermore, given the real-life approach of our study, we gathered data from several pathology centres, and some missing data could have occurred, especially concerning smoking habits. Nevertheless, our heterogeneous population encompasses all consecutive patients we could retrieve, without any selection except histology and exploitable tissue, thus reducing possible bias. Moreover, all our samples were analyzed by the same molecular platform within a relatively short time span, thus minimizing technical differences related to equipment or manpower.

In conclusion, we show interesting and clinically relevant variations of activating somatic mutations that are targetable in subgroups of patients according to the gene and patient's birthplace. In particular, African women exhibit molecular profiles in their tumor closer to those of Asian than Caucasian women, and they could dramatically benefit from anti-*EGFR* and anti-*ERBB2* targeted therapies.

## PATIENTS AND METHODS

This study was conducted in France during daily clinical practice for somatic EGFR mutations research and involved clinicians, pathologists and biologists. Clinical and biological dataavailable in patients’ medical records werecollected by the physician in charge of the patient. All the data were centralized in a database and made completely anonymous. The database has been declared to the CNIL (Commission nationale de l'informatique et des libertés; French dataprotection authority).

The study was conducted on a total of 2,240 consecutive patients with newly-diagnosed, histologically-proven metastatic or recurrent NSCLC and tested for EGFR mutational status in the routine clinical setting using the same somatic genetic testing strategy, between November 2011 and December 2013.

Mutational testing was performed at a single platform with expertise in both molecular and pathological diagnostics. The platform was ISO 15189 (for screening and identification of mutations in EGFR, KRAS and BRAF) and INCa (French National Institute of Cancer) certified. ALK or ROS1 translocations were not included in the analysis.

Age, gender and birthplace, collected by physicians at the first visit, were mentioned in the medical record of each patient. When birthplace was not specified in the medical record, it was found out from the personal identity number at the French Health care system (Sécurité sociale). For African or Asian descendants who were born in France, parents’ birthplace was considered when mentioned in the medical record. If this information was not available, patients were excluded from the study. Thus, full data were available for a total of 2,219 patients (out of 2,240), which were included in the analysis.

Patients were classified according tosSmoking status as never smokers, former smokers (≥ 5 years of quitting) or active smokers (including recent quitters, light or heavy smokers, with a cut off at 10 pack/years) and the number of pack/years of smoking (one pack/year is defined as 20 cigarettes per day during 1 year) was retrieved [50, 51].

Histology was assessed by specialist lung cancer pathologists using the WHO criteria, and adenocarcinoma described according to the International Association for the Study of Lung Cancer classification [52]. Histological type and grading was defined using haematoxylin and eosin (HE)-stained biopsies after serial sectioning. Immunohistochemistry used the recommended panel of antibodies for classification of NSCLC (TTF1, CK7, CK5/6 and/or p63). Clinico-pathological stage was assigned according to the 7^th^ edition of the tumor-node-metastasis classification [53]. Squamous cell carcinoma samples were excluded from the study.

Mutational status analysis was performed using formalin-fixed and paraffin-embedded tumours containing more than 10% of cancer cells. DNA was extracted after overnight paraffin digestion at 56°C. All the known activation and resistance gene abnormalities in *EGFR* exons 18 to 21, *KRAS* exons 2 and 3, *BRAF* exons 11 and 15, *ERBB2* exon 20 and *PI3KCA* exons 9 and 20 were screened. Genetic abnormalities were analysed using a combination of technologies. The screening of these gene abnormalities was performed for each exon using a High Resolution Melting (HRM) technology (Lightcycler 480, Roche), together with a concomitant search by allelic discrimination for the 7 most frequent gene abnormalities in *KRAS* exons 2 and 3; *EGFR* (L858R) and *BRAF* (V600E). Base pairs deletion in exon 19 of *EGFR* were analysed by fragment analysis (ABI 310, Applied). When gene abnormalities, especially uncommon mutations, were not observed by HRM, but only using the other techniques of identification, a Sanger sequencing was performed.

All sequence analyses were checked for plausibility. All techniques are broadly accepted for routine detection of *EGFR* mutational status. The laboratory of molecular pathology successfully completed the round robin test of the French quality initiative certified Societies [54]. It also got the ISO 15189 certification in 2013 for the screening and the identification of the *EGFR*, *KRAS* and *BRAF* mutations.

### Statistical analysis

We used percentages for qualitative variables and mean and standard deviations for quantitative variables. Multivariate binary logistic regression was used to determine the independent predictive factors for the occurrence of specific mutations. In case of missing data (especially for smoking status), cases were excluded from the model. Differences in clinical characteristics according to ethnic origin were explored using Student's t or chi-squared tests. Significance was determined by a P-value < 0.05. All statistics were performed using SPSS software (SPSS version 17.0 for Windows, IBM Inc., Chicago, IL, USA).

## Abbreviations

NSCLC: non-small cell lung cancer
EGFR: epidermal growth factor receptor
ERBB2: human epidermal growth factor receptor 2 (avian erythroblastosis oncogene B2 homolog)
KRAS: Kristen rat sarcoma oncogene homolog
BRAF: murine sarcoma viral oncogene B homolog
PI3KCA: phosphatidylinositol-4, 5-bisphosphate 3-kinase catalytic subunit alpha.
